# Involvement of ADAM17-Klotho Crosstalk in High Glucose-Induced Alterations of Podocyte Function

**DOI:** 10.3390/ijms26020731

**Published:** 2025-01-16

**Authors:** Dorota Rogacka, Patrycja Rachubik, Marlena Typiak, Tomasz Kulesza, Irena Audzeyenka, Moin A. Saleem, Honorata Sikora, Natalia Gruba, Magdalena Wysocka, Adam Lesner, Agnieszka Piwkowska

**Affiliations:** 1 Laboratory of Molecular and Cellular Nephrology, Mossakowski Medical Research Institute, Polish Academy of Sciences, 80-308 Gdansk, Poland; prachubik@imdik.pan.pl (P.R.); t.kulesza@gumed.edu.pl (T.K.); iaudzeyenka@imdik.pan.pl (I.A.); apiwkowska@imdik.pan.pl (A.P.); 2 Department of General and Medical Biochemistry, Faculty of Biology, University of Gdansk, 80-309 Gdansk, Poland; marlena.typiak@ug.edu.pl; 3 Laboratory of Molecular Enzymology and Oncology, Intercollegiate Faculty of Biotechnology, University of Gdansk and Medical University of Gdansk, 80-210 Gdansk, Poland; 4 Bristol Renal, University of Bristol, Dorothy Hodgkin Building, Bristol BS1 3NY, UK; m.saleem@bristol.ac.uk; 5 Department of Biomedical Chemistry, Faculty of Chemistry, University of Gdansk, 80-308 Gdansk, Poland; honorata.flemming@phdstud.ug.edu.pl (H.S.); magdalena.wysocka@ug.edu.pl (M.W.); 6 Department of Environmental Technology, Faculty of Chemistry, University of Gdansk, 80-308 Gdansk, Poland; natalia.gruba@ug.edu.pl (N.G.); adam.lesner@ug.edu.pl (A.L.)

**Keywords:** ADAM17, Klotho, high glucose, filtration barrier, oxidative stress, podocytes

## Abstract

Microalbuminuria is the earliest clinical abnormality in diabetic kidney disease. High glucose (HG) concentrations are associated with the induction of oxidative stress in podocytes, leading to disruption of the glomerular filtration barrier. Our recent study revealed a significant decrease in the membrane-bound fraction of Klotho in podocytes that were cultured under HG conditions. Given that disintegrin and metalloproteinase 17 (ADAM17) is responsible for the shedding of Klotho from the cell membrane, the present study investigated the impact of HG on the interplay between ADAM17 and Klotho in human podocytes. We demonstrated that ADAM17 protein levels significantly increased in urine, renal tissue, and glomeruli from diabetic rats, with a concomitant increase in glomerular albumin permeability. High glucose increased ADAM17 extracellular activity, NADPH oxidase activity, and albumin permeability in podocytes. These effects were reversed after treatment with ADAM17 inhibitor, in cells with downregulated ADAM17 expression, or after the addition of Klotho. Additionally, elevations of extracellular ADAM17 activity were observed in podocytes with the downregulation of Klotho expression. Our data indicate a novel mechanism whereby hyperglycemia deteriorates podocyte function via ADAM17 activation. We also demonstrated the ability of Klotho to protect podocyte function under hyperglycemic conditions in an ADAM17-dependent manner.

## 1. Introduction

The unique structure and function of podocytes are essential for maintaining proper kidney function. These highly specialized cells play a key role in regulating the glomerular filtration barrier. Podocyte foot processes interdigitate with each other, leaving narrow slit diaphragms between them. Slit diaphragms prevent the passage of large molecules, such as proteins and blood cells, into urine while allowing smaller molecules to pass through [1]. This selective filtration is essential for maintaining blood composition and preventing proteinuria. Slit diaphragms can adapt and change their permeability in response to various physiological and pathological conditions.

Podocyte injury that leads to albuminuria is a characteristic feature of diabetic nephropathy, also known as diabetic kidney disease (DKD), which is a leading cause of kidney disease worldwide. The earliest detectable clinical abnormality in DKD is microalbuminuria, which is often considered a warning sign that diabetes may be affecting the kidneys. Early events during the development of DKD also include an increase in glomerular filtration (hyperfiltration). These phenomena are both closely related to glomerular injury, a hallmark of DKD. Microalbuminuria and proteinuria are currently used as indicators of kidney damage and DKD, but ongoing research is being conducted to discover more sensitive and specific markers. Disintegrin and metalloproteinase 17 (ADAM17) could be considered a potential marker of renal dysfunction, in which an increase in urinary ADAM17 levels has been observed in diabetes patients [2].

ADAM17 is a transmembrane protein that belongs to the ADAM family of enzymes. ADAMs are involved in the proteolytic shedding of cell surface proteins, including cytokines, growth factors, receptors, and adhesion molecules. This shedding process has significant implications for cell signaling, immune responses, and various physiological processes. ADAM17 is a type I transmembrane protein that exists in two primary isoforms: a full-length, inactive form with the prodomain serving as an inhibitory element. Prodomain removal is a crucial step in the regulation of ADAM17’s shedding activity [3]. ADAM17 and its relative ADAM10 are considered principal ADAM sheddases that are widely expressed in mammalian cells. ADAM17 substrates include tumor necrosis factor-α and various epidermal growth factor receptor ligands, including amphiregulin, epiregulin, heparin-binding epidermal growth factor-like growth factor, and transforming growth factor α, all of which are cleaved and processed by ADAM17 [4]. A result of ADAM17-dependent cleavage is the release of soluble forms of these proteins into the extracellular space. An important substrate of ADAM17 is Klotho, a transmembrane protein that is primarily expressed in the kidneys [5] and plays a critical role in regulating various aspects of kidney function, including phosphate metabolism and electrolyte balance [6]. Kidney cells have been identified as the primary source of the soluble form of Klotho (sKlotho) in the circulation, which is considered to function as a hormone with beneficial effects on various target tissues [7], including antioxidative stress [8] and antiinflammatory actions [9]. Furthermore, high levels of sKlotho have been associated with a lower risk of renal dysfunction [10]. Studies have shown that sKlotho levels decline with advancing stages of chronic kidney disease [11]. A more recent study showed that low levels of sKlotho in patients with type 2 diabetes predicted renal function decline and may be a biomarker of renal dysfunction [12]. In turn, in animal studies, Klotho overexpression reduced oxidative stress, renal cell hypertrophy, inflammation, and apoptosis [13,14]. Klotho expression has been detected in various regions of the kidney, including cells of proximal and distal tubules of the nephron, human glomeruli, and mouse podocytes [15,16].

Accumulating evidence confirms that high glucose (HG) concentrations affect podocyte metabolism and function [17]. Oxidative stress plays a critical role in the initiation and progression of DKD. It results from the overproduction of reactive oxygen species (ROS) with the concomitant insufficient activity of antioxidant pathways. Our earlier study found that the exposure of podocytes to HG concentrations caused oxidative stress, reflected by an increase in ROS production and NADPH oxidase activity, with an important role of the NADPH oxidase 4 (NOX4) subunit in ROS elevation and regulation of antioxidant enzymes in these cells [18]. NADPH oxidase is the main source of ROS in podocytes cultured in an HG milieu [18]. NOX4 is expressed at relatively high levels in the kidney. In the context of DKD, NOX4 is one of the most extensively studied isoforms of NADPH oxidase subunits, which is often upregulated [19]. We also found that HG attenuated the insulin responsiveness of podocytes [20] and increased permeability to albumin via the podocyte monolayer in vitro [21]. Our recent study demonstrated that Klotho levels increased in serum and decreased in renal tissue, glomeruli, and urine in diabetic rats. The increased shedding of Klotho under hyperglycemic conditions caused a decrease in the amount of podocyte membrane-bound Klotho. Moreover, the addition of Klotho impacted glucose metabolism in podocytes and albumin permeability of the glomerular filtration barrier under HG conditions [22].

ADAM17 is responsible for the shedding of Klotho from the cell membrane, and studies have demonstrated that HG upregulates and activates ADAM17 in mesangial cells [23,24]. An HG-induced increase in ROS production may impede the normal function of ADAM17 in the proteolytic release of Klotho. This interference can potentially impair the cleavage process, resulting in reduced levels of Klotho. The main objective of the present study was to examine the effect of hyperglycemic conditions on ADAM17-Klotho crosstalk in human podocytes. Given that ADAM17 is involved in the modulation of NADPH oxidase activity in the kidney and that higher ADAM17 expression and activity in association with NOX4 upregulation were observed in mouse proximal tubular cells in a hyperglycemic environment [25], we examined the effects of ADAM17 modulation on oxidative stress induction in podocytes that were exposed to HG concentrations. We also investigated whether Klotho protects podocyte function under hyperglycemic conditions in an ADAM17-dependent manner.

## 2. Results

### 2.1. ADAM17 Levels Are Increased in Renal Tissues in Rats with STZ-Induced Diabetes

**Figure 1:**
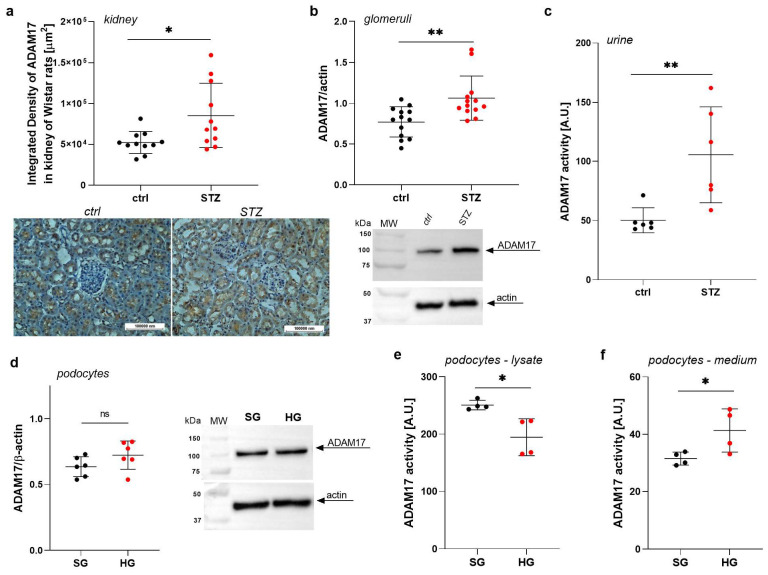
Elevation of ADAM17 protein levels and activity in renal tissues and urine in healthy rats and rats with STZ-induced diabetes and effects of high glucose concentrations on ADAM17 activity in podocytes. (**a**) Increase in ADAM17 protein levels (brown staining) in renal tissues (*n* = 11). Scale bar = 100 μm. (**b**) Increase in ADAM17 protein levels in diabetic glomeruli (*n* = 13). (**c**) ADAM17 activity (*n* = 6) was elevated in urine in diabetic rats. (**d**) ADAM17 protein expression in podocytes (*n* = 6). (**e**) Decrease in ADAM17 activity in podocyte lysates (*n* = 4). (**f**) Increase in extracellular ADAM17 activity in podocytes (*n* = 4). The results are expressed as the mean ± SD. ** *p* < 0.01, * *p* < 0.05. Ctrl, control; ns, non-statistically significant; STZ, streptozotocin; SG, standard glucose; HG, high glucose.

In the present study, we investigated whether ADAM17 may play a role in the pathogenesis of diabetic kidney disease via the induction of glomerular injury. For this purpose, we used rats with diabetes induced by STZ compared with age-matched control healthy rats. We conducted immunohistochemical staining of kidney tissue samples that were obtained from both STZ-treated Wistar rats and control rats. The density of brown staining in the stained tissue sections directly correlated with the amount of ADAM17 protein. There was a significant 56% increase in ADAM17 levels in the renal tissue (*p* = 0.0154; Figure 1a). We also analyzed lysates that were derived from glomeruli that were isolated from STZ-treated rats to assess the changes in the amount of ADAM17 protein. The isolated glomeruli from STZ-treated rats exhibited a 38% increase in ADAM17 levels (*p* = 0.0029; Figure 1b). These findings suggest that ADAM17 protein levels are significantly elevated in both renal tissue and isolated glomeruli from STZ-treated rats, implying a potential link between ADAM17 and injury in glomeruli, which is often associated with diabetes.

### 2.2. ADAM17 Levels and Activity Are Elevated in the Urine of Rats with STZ-Induced Diabetes

Since excessive ADAM17 activity may lead to detrimental renal effects, we measured ADAM17 activity in urine to examine whether it is released from the kidneys as a response to kidney injury or dysfunction in diabetes. We found a greater-than-two-fold increase in ADAM17 activity in the urine of STZ-treated rats (*p* = 0.0043; Figure 1c), measured using a specific peptide substrate designed and optimized for ADAM17 [26]. Our results suggest that the increase in ADAM17 activity in urine may be associated with kidney injury in diabetes. Since urine is a valuable source of biomarkers reflecting kidney function and health, measuring ADAM17 activity in urine could serve as a potential biomarker for kidney injury or dysfunction, particularly in the context of diabetes.

### 2.3. High Glucose Concentrations Impact the Extracellular Activity of ADAM17 in Podocytes

We analyzed ADAM17 protein expression and activity in lysates obtained from podocytes cultured in SG or HG media for 5 days, as well as in the extracellular medium. We detected ADAM17 protein expression in podocytes and observed a non-statistically significant increase in the ADAM17 protein levels in these cells in response to high glucose concentrations (Figure 1d). Additionally, we observed a 32% decrease in ADAM17 activity in the cell lysates (*p* = 0.0149; Figure 1e) and a 31% increase in extracellular ADAM17 activity (*p* = 0.0149; Figure 1f).

### 2.4. Inhibition of ADAM17 Improves Albumin Permeability Across the Glomerular Filtration Barrier in STZ Rats and Across a Monolayer of Podocytes Exposed to HG Concentrations

**Figure 2:**
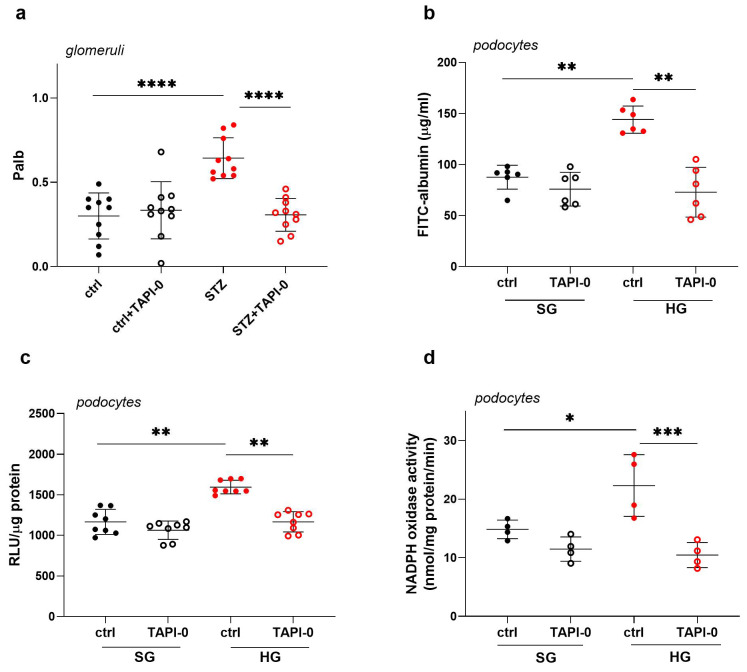
Inhibition of ADAM17 improved albumin permeability in the glomeruli of diabetic rats and across the monolayer of podocytes exposed to high glucose concentrations, with concomitant attenuation of HG-induced oxidative stress in podocytes. (**a**) ADAM17 inhibition in the presence of TAPI-0 improved glomerular albumin permeability (*n* = 10). (**b**) ADAM17 inhibition ameliorated albumin permeability via the monolayer of podocytes (*n* = 6) that were cultured in the presence of HG concentrations. (**c**) The elevation of ROS production in podocytes that were exposed to HG was reduced in the presence of TAPI-0 (*n* = 8). (**d**) The induction of oxidative stress under HG conditions, manifested by an increase in NADPH oxidase activity, was attenuated by the ADAM17 inhibitor TAPI-0 (*n* = 4). The results are expressed as the mean ± SD. **** *p* < 0.0001, *** *p* < 0.001, ** *p* < 0.01, * *p* < 0.05. Ctrl, control; STZ, streptozotocin; SG, standard glucose; HG, high glucose; TAPI-0, ADAM17 inhibitor.

As ADAM17 activity was significantly increased in the urine of diabetic rats and in the extracellular medium of podocytes exposed to high glucose concentrations, the next step was to examine the effect of ADAM17 on the filtration function of both glomeruli and podocytes using the ADAM17 inhibitor TAPI-0. We measured albumin permeability (P_alb_) in glomeruli that were isolated from healthy control rats and STZ rats. Under diabetic conditions, we observed a significant, greater than two-fold increase in glomerular P_alb_ (*p* < 0.0001; Figure 2a). In the presence of the ADAM17 inhibitor TAPI-0 (10 µM, 20 min), the albumin permeability of glomeruli that were isolated from STZ rats significantly decreased by 55% (*p* < 0.0001; Figure 2a) and reached levels that were comparable to glomeruli that were isolated from healthy rats. These findings indicate that ADAM17 inhibition with TAPI-0 has a positive impact on reducing albumin permeability in glomeruli in diabetic rats, potentially suggesting a promising therapeutic approach to counteract kidney damage in the context of diabetes-related renal complications. Next, we examined the effects of TAPI-0 treatment on the permeability to albumin of a monolayer of podocytes that were cultivated under hyperglycemic conditions. Consistent with our previous reports [21], the cells that were exposed to HG concentrations exhibited a significant 1.6-fold increase in albumin flux via the podocyte monolayer (*p* = 0.0022; Figure 2b). The incubation of HG-exposed cells with TAPI-0 (10 µM, 24 h) decreased albumin permeability by 49% (*p* = 0.0022; Figure 2b), effectively restoring permeability to levels that were similar to the control conditions. These results suggest potential therapeutic implications of inhibiting ADAM17 in the context of protecting integrity of the glomerular filtration barrier in the kidneys.

### 2.5. ADAM17 Inhibition Decreases HG-Induced Oxidative Stress in Podocytes

Elevated oxidative stress is both a key cause and consequence of diabetes, and it is believed to drive the cellular changes that contribute to diabetic complications. Our previous studies demonstrated that HG concentrations induced ROS production via the activation of NADPH oxidase [18]. It was also demonstrated that ADAM17 activation resulted in increased NADPH oxidase activity in the kidney cortex of diabetic mice [25]. Thus, in the next series of experiments, we examined the effect of ADAM17 on oxidative stress induction in podocytes. We cultured podocytes under SG or HG conditions in the presence of TAPI-0 (10 µM, 24 h). According to our previous studies, HG concentrations induced ROS production via the activation of NADPH oxidase. In the presence of TAPI-0, the HG-induced elevation of ROS levels decreased by 27% (*p* = 0.0085; Figure 2c). ADAM17 inhibition with TAPI-0 also reduced NADPH activity by 53% (*p* = 0.0005; Figure 2d) in HG-cultured cells, restoring it to control levels. This suggests that ADAM17 may be involved in the deterioration of podocyte function by participating in the oxidative stress effects induced by hyperglycemia.

### 2.6. ADAM17 Downregulation Exerts a Beneficial Effect on the Function of Podocytes Under High Glucose Conditions

**Figure 3:**
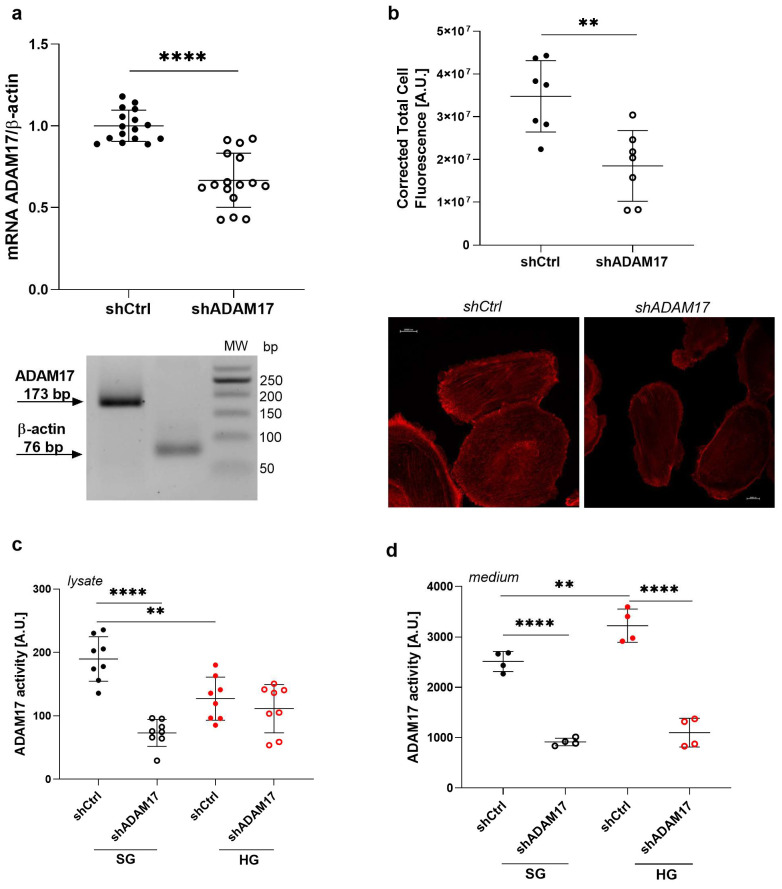
Silencing of ADAM17 gene expression attenuates the increase in its extracellular activity in podocytes exposed to HG medium. (**a**) ADAM17 mRNA levels decreased in shADAM17 cells (*n* = 16). A representative agarose gel of PCR products is shown. (**b**) Analysis of immunofluorescence staining of ADAM17 in control podocytes (shCtrl) and cells with downregulated ADAM17 expression (shADAM17). The corrected total cell fluorescence was measured using ImageJ 4.1 software (*n* = 7–11). Scale bar = 20 μm. (**c**,**d**) ADAM17 activity in (**c**) the lysate (*n* = 8) and (**d**) extracellular medium (*n* = 4) of shADAM17 cells compared with shCtrl cells that were cultured under SG or HG conditions. The results are expressed as the mean ± SD. **** *p* < 0.0001, ** *p* < 0.01. SG, standard glucose; HG, high glucose.

Since TAPI-0 is a non-selective inhibitor of ADAM17 and also inhibits matrix metalloproteinases (MMPs) [27], we transfected podocytes with short-hairpin RNA targeting ADAM17 (shADAM17) to downregulate its expression and investigate whether the effect of high glucose concentrations on the permeability of the podocyte monolayer to albumin depends on ADAM17. A decrease in ADAM17 expression was confirmed through real-time PCR (33% decrease in ADAM17 mRNA levels; *p* < 0.0001; Figure 3a), and immunofluorescent staining (46% decrease; *p* = 0.0032; Figure 3b). Additionally, ADAM17 downregulation decreased its activity in both the lysate and medium under SG conditions by 61% (*p* < 0.0001; Figure 3c) and 64% (*p* < 0.0001; Figure 3d), respectively. We found that ADAM17 activity in cell lysates from shCtrl cells after exposure to an HG medium decreased by 33% (*p* = 0.0028; Figure 3c) but increased in the extracellular medium by 28% (*p* = 0.0041; Figure 3d). ADAM17 downregulation did not further affect ADAM17 activity in lysates from shADAM17 cells compared with the shCtrl under HG conditions (Figure 3c) but significantly decreased ADAM17 activity by 66% in the medium (*p* < 0.0001; Figure 3d).

**Figure 4:**
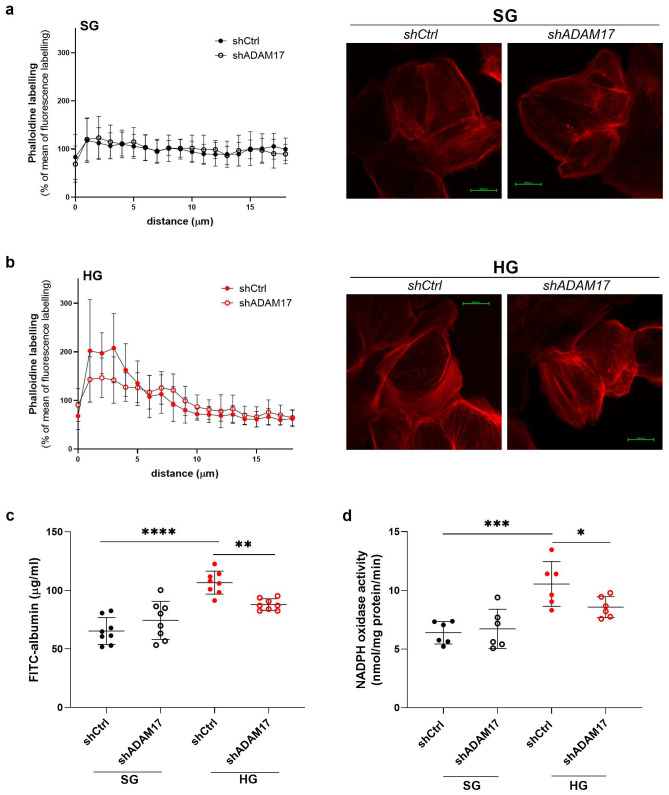
ADAM17 downregulation remodels the actin cytoskeleton in podocytes and improves albumin permeability through the monolayer of podocytes cultured under high glucose conditions. The F-actin network was labeled with isothiocyanate phalloidin and visualized by fluorescence microscopy. Control cells (shCtrl) and cells with the silencing of ADAM17 gene expression (shADAM17) were grown on coverslips and incubated in (**a**) standard glucose (SG; 11 mM) or (**b**) high glucose (HG; 30 mM) medium. Scale bar = 25 μm. Digitized fluorescence images of the F-actin network were used to generate fluorescence intensity profiles (from the basal membrane to the nucleus) using NIS-Elements 5.11.02 imaging software (*n* = 20). (**c**) Effects of ADAM17 downregulation on albumin permeability through the monolayer that was formed by shCtrl or shADAM17 podocytes that were cultured in an SG or HG environment (*n* = 8). (**d**) The high glucose-induced activation of NADPH oxidase was attenuated in shADAM17 podocytes (*n* = 6). The results are expressed as the mean ± SD. **** *p* < 0.0001, *** *p* < 0.001, ** *p* < 0.01, * *p* < 0.05. SG, standard glucose; HG, high glucose.

Our previous study showed that the high glucose-induced increase in albumin permeability across the podocyte monolayer is linked to rearrangement of the podocyte cytoskeleton [28]. To elucidate whether ADAM17 downregulation impacts organization of the actin cytoskeleton, we examined the F-actin distribution pattern in shADAM17 podocytes that were cultured in SG or HG medium compared with shCtrl cells. The downregulation of ADAM17 did not affect the intracellular F-actin distribution in cells that were cultured under SG conditions (Figure 4a). High glucose concentrations significantly modified intracellular F-actin localization in shCtrl podocytes, but ADAM17 downregulation partially reversed the changes in F-actin distribution to a pattern that was similar to that of shCtrl cells (Figure 4b). These results suggest that ADAM17 downregulation may play a role in preserving normal organization of the actin cytoskeleton in podocytes under hyperglycemic conditions, which could be relevant in the context of diabetes where hyperglycemia can impact kidney function.

High glucose concentrations increased albumin permeability by 64% in the monolayer of shCtrl cells (*p* < 0.0001; Figure 4c). However, in the shADAM17 cells, the increase in albumin permeability decreased by 18% (*p* = 0.0078; Figure 4c). These results indicate that although ADAM17 downregulation in HG-cultured cells did not affect its activity within the cell lysate, it significantly reduced ADAM17 activity in the extracellular medium and mitigated the HG-induced increase in albumin permeability in podocytes. Additionally, we examined the NADPH oxidase activity in podocytes with the silencing of *ADAM17* gene expression to confirm that the previously observed effect of TAPI-0 was indeed ADAM17-dependent. In the shCtrl podocytes, HG increased NADPH oxidase activity by 65% (*p* = 0.0008; Figure 4d), whilst in the HG-exposed podocytes with ADAM17 downregulation, NADPH activity decreased by 19% (*p* = 0.0454; Figure 4d). These results suggest that ADAM17 downregulation may have a beneficial effect on podocyte function under HG conditions.

### 2.7. Klotho Deficiency Enhances ADAM17 Activity in the Extracellular Medium of Podocytes

**Figure 5:**
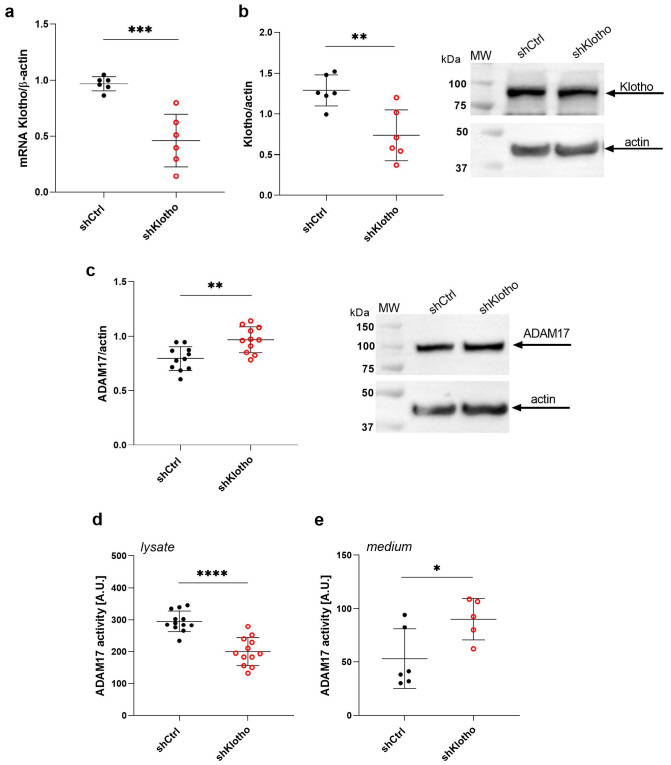
The suppression of Klotho gene expression promotes the shedding of ADAM17 in podocytes. (**a**) mRNA (*n* = 6) and (**b**) protein (*n* = 6) levels of Klotho in shKlotho podocytes. (**c**) ADAM17 protein levels in shKlotho podocytes (*n* = 11). (**d**,**e**) Activity of ADAM17 in (**d**) shKlotho podocyte lysates (*n* = 12) and (**e**) extracellular medium (*n* = 5–6). The results are expressed as the mean ± SD. **** *p* < 0.0001, *** *p* < 0.001, ** *p* < 0.01, * *p* < 0.05.

Numerous studies have shown that Klotho deficiency is strongly associated with the pathogenesis of diabetic kidney disease [29]. Our previous study demonstrated that the shedding of soluble Klotho was increased in hyperglycemia and caused a decrease in the amount of podocyte membrane-bound Klotho, affecting podocyte filtration functions [22]. Additionally, our recent study revealed that Klotho deficiency in podocytes induced oxidative stress and increased ADAM10 activity in an extracellular medium with a concomitant increase in albumin permeability through an shKlotho podocyte monolayer [30]. Thus, in the next step of our study, we silenced *Klotho* gene expression in podocytes by lentiviral transduction of Klotho shRNA and examined the ADAM17 protein levels in shKlotho podocytes, and the ADAM17 activity in both the shKlotho cell lysate and extracellular medium. A decrease in Klotho expression was confirmed through real-time PCR (52% decrease in Klotho mRNA levels; *p* = 0.0005; Figure 5a), and Western blot (43% decrease in Klotho protein levels; *p* = 0.0042; Figure 5b). *Klotho* gene silencing caused a 22% increase in ADAM17 protein levels (*p* = 0.002; Figure 5c), decreased ADAM17 activity by 32% in the shKlotho podocytes (*p* < 0.0001; Figure 5d), and significantly increased ADAM17 activity by 70% in the extracellular medium (*p* = 0.0340; Figure 5e). These findings indicate that the loss of Klotho promotes the shedding of ADAM17 in podocytes and supports the link between Klotho and ADAM17 in the context of podocyte function.

### 2.8. Klotho Attenuates the HG-Induced Increase in ADAM17 Extracellular Activity in Podocytes

**Figure 6:**
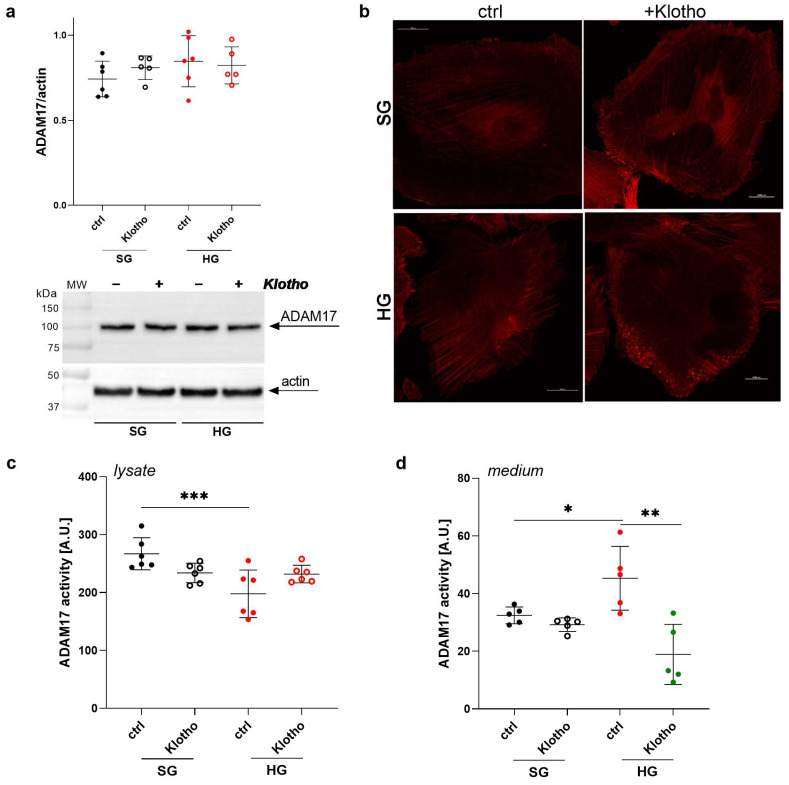
Klotho reduces the HG-induced elevation of ADAM17 extracellular activity in podocytes. (**a**) ADAM17 protein levels (*n* = 5–6) and (**b**) intracellular distribution in podocytes that were cultured under SG or HG conditions in the presence of Klotho. Scale bar = 25 μm. (**c**,**d**) Activity of ADAM17 in (**c**) podocyte lysates (*n* = 6) and (**d**) the extracellular medium (*n* = 5) in the presence of Klotho during exposure to an HG environment. The results are expressed as the mean ± SD. *** *p* < 0.001, ** *p* < 0.01, * *p* < 0.05. SG, standard glucose; HG, high glucose.

The administration of Klotho has been found to be an effective treatment for kidney injury and the preservation of renal function in early clinical studies [31,32]. Our previous study demonstrated a positive effect on maintaining proper glomerular permeability in diabetic rats and albumin permeability in a podocyte monolayer under hyperglycemic conditions, confirming that Klotho exerts renal protective effects [22]. In this study, we examined whether the deteriorative effects of increased extracellular ADAM17 activity on podocyte function may be reversed by Klotho administration. No significant changes in ADAM17 protein levels were observed in podocytes that were cultured in an SG or HG environment in the presence of Klotho (Figure 6a). However, we detected Klotho-induced changes in the intracellular distribution of ADAM17 protein under both SG and HG conditions. In the presence of Klotho, ADAM17 protein was located closer to the cell membrane and appeared as scattered granules, especially in cells that were cultured in the HG environment (Figure 6b).

In the presence of Klotho, podocytes that were cultured under HG conditions exhibited a non-significant increase in ADAM17 activity in cell lysates (Figure 6c) and a significant decrease in ADAM17 activity in the extracellular medium (58%; *p* = 0.0046; Figure 6d). These results suggest that ADAM17 activity modulation in response to Klotho may play an important role in regulating albumin permeability through a monolayer of podocytes under hyperglycemic conditions.

## 3. Discussion

The main findings of the present study were that (i) HG concentrations increase the extracellular activity of ADAM17, leading to oxidative stress and detrimental effects on podocyte function; (ii) inhibition or downregulation of ADAM17 tightens the podocyte filtration barrier and reduces HG-induced oxidative stress; (iii) Klotho deficiency is associated with increased extracellular activity of ADAM17, accompanied by higher albumin permeability through the podocyte monolayer; and (iv) Klotho plays a protective role against the ADAM17-dependent deleterious effects in HG-exposed podocytes. These findings highlight the complex interplay between HG levels, ADAM17 activation, Klotho deficiency, and podocyte function.

An increase in ADAM17 expression was previously shown to be positively associated with the development of insulin resistance [33]. This early study demonstrated that ADAM17 activity increased in skeletal muscle in insulin-resistant obese diabetic patients. Insulin signaling to podocytes is critical for proper function of the filtration barrier [34]. Our research demonstrated that podocytes that are exposed to a hyperglycemic environment lose insulin responsiveness [35], and the function of these cells is altered, reflected by an increase in permeability to albumin via the podocyte monolayer, accompanied by F-actin distribution to the cortical regions of podocytes [28]. Our recent results showed that the incubation of rat glomeruli and a monolayer of human podocytes with recombinant Klotho significantly decreased albumin permeability under HG conditions and that HG concentrations decreased the levels of the membrane form of Klotho protein but increased sKlotho shedding in podocytes [22]. Moreover, we demonstrated that Klotho downregulation increased albumin permeability and oxidative stress in podocytes [30].

These findings raise the hypothesis that the observed changes in Klotho protein levels and distribution in podocytes could be related to an increase in the expression of its sheddase ADAM17 in podocytes that are exposed to hyperglycemia or in diabetic models. ADAM17 appears to be a likely factor in the altered distribution of Klotho in podocytes. Klotho exists in both membrane-bound and soluble forms, and ADAM17 activity could directly impact the balance between these forms, which has consequences for the function and integrity of podocytes and the glomerular filtration barrier. Indeed, the present study confirmed the deleterious effect of HG concentrations on the glomerular filtration barrier and demonstrated that this effect may be associated with changes in the interaction between ADAM17 and Klotho in podocytes. We showed that ADAM17 protein levels and activity are elevated in renal tissues and urine in diabetic rats. These results are consistent with findings in patients with diabetes who exhibit an increase in urinary ADAM17 levels [2]. Our observations also align with previous findings that ADAM17 protein expression increased in the kidneys in *db*/*db* mice, which was associated with the lower renal expression of an endogenous inhibitor of ADAM17, inhibitor of metalloproteinase 3 (TIMP3), leading to the exacerbation of DKD [36]. In smooth muscle cells, the inhibition of the activity and levels of deacetylase sirtuin 1 (SIRT1) reduced TIMP3 expression, whereas SIRT1 overexpression increased the activity of the TIMP3 promoter [37]. SIRT1 activity is associated with the regulation of insulin responsiveness in podocytes, and its protein levels and activity decrease under hyperglycemic conditions [38]. Thus, this mechanism of ADAM17 regulation in these cells cannot be excluded.

We demonstrated that high ADAM17 activity in the extracellular medium could cause a worsening of glomerular filtration barrier function, indicated by its tightening after ADAM17 inhibition by TAPI-0 and after silencing the *ADAM17* gene expression in podocytes. ADAM17 downregulation reversed the F-actin patterns, which were changed in the HG-exposed podocytes, causing them to resemble control podocytes. Studies showed that Klotho deficiency in diabetes was associated with podocyte apoptosis, podocyte injury, and proteinuria [39]. The present study found that Klotho deficiency in podocytes with the silencing of *Klotho* gene expression increased the ADAM17 protein levels, decreased its activity in cell lysates, and increased its activity in the extracellular medium in these cells. These changes may lead to an increase in albumin permeability through the monolayer of shKlotho podocytes, similar to the results observed previously for ADAM10 [30].

In the present study, we examined the effects of ADAM17-Klotho interactions on oxidative stress in podocytes under hyperglycemic conditions. In cardiomyocytes, NOX4 was found to control the transcription and translation of ADAM17, and ROS were required in the NOX4-mediated upregulation of ADAM17 expression [40]. Conversely, ADAM17 was suggested to be an upstream mediator of NADPH oxidase in the diabetic kidney. In cultured proximal tubular epithelial cells, HG concentrations activated ADAM17 and increased NOX4 protein expression and NADPH oxidase activity [25]. Our results demonstrated that ADAM17 inhibition with TAPI-0 or *ADAM17* gene silencing reduced the activity of NADPH oxidase, decreasing HG-induced oxidative stress in podocytes. These results were consistent with the findings in diabetic OVE26 mice, demonstrating that ADAM17 inhibition led to a reduction in ROS production through the downregulation of NOX4, suggesting its role in the induction of oxidative stress and kidney injury [25]. Additionally, our recent study demonstrated that inhibiting ADAM10 with GI254023x reduced NADPH oxidase activity and inhibited ROS production [30]. This suggests that the activation of these sheddases, due to exposure to the HG milieu, may contribute to the impairment of podocyte function through the induction of oxidative stress.

Klotho has been shown to protect cells against oxidative stress [41], and its administration has been found to reduce oxidative stress in *db*/*db* mice [42]. It was shown that the adenovirus-mediated overexpression of Klotho significantly reduced oxidative stress in podocytes cultured under high glucose conditions and in the kidneys of *db*/*db* mice [43]. The Klotho-dependent suppression of NOX2 expression, leading to the attenuation of oxidative stress, was also found in rat aortic smooth muscle cells and might be mediated by the cyclic adenosine monophosphate-protein kinase A pathway [44]. Klotho protein also decreased the NOX2 and NOX4 levels in human cardiomyocytes [45]. Our previous study revealed that Klotho deficiency increases ROS production with a concomitant rise in NOX4 protein expression and NADPH oxidase activation, which results in increased permeability to albumin across the podocyte monolayer [30]. Thus, our results suggest that Klotho plays a critical role in protecting podocytes against the deteriorating effects of oxidative stress that is induced by HG and that the ability of Klotho to counteract the damaging effects of oxidative stress in podocytes is essential for maintaining their function and glomerular filtration barrier integrity in the kidneys.

**Figure 7:**
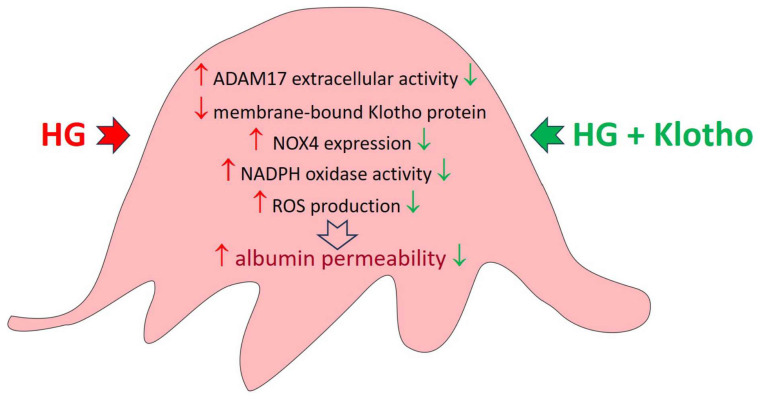
Proposed mechanism of the ADAM17-dependent detrimental effect on podocyte function induced by high glucose concentrations.

In summary, the present results suggest that ADAM17 activation, driven by HG and leading to Klotho deficiency in podocytes, can induce oxidative stress and deteriorate podocyte function (Figure 7). The protective role of Klotho in reducing oxidative stress and the potential benefit of ADAM17 inhibition in this context were also noted. The precise mechanisms of crosstalk between ADAM17 and Klotho in the context of podocyte function under hyperglycemic conditions are still not fully understood, but our findings have implications for understanding the interplay between ADAM17 and Klotho in podocytes under hyperglycemic conditions and provide insights into the pathophysiology of kidney-related disorders, particularly those associated with diabetes and its renal complications.

## 4. Materials and Methods

### 4.1. Ethical Approval

All experiments adhered to the guidelines outlined in EU Directive 2010/63/EU for animal experimentation. The protocol was approved by the Local Bioethics Commission in Bydgoszcz, Poland (protocol number 51/2018, date of approval 14 December 2018).

### 4.2. Experimental Animals and Metabolic Cage Studies

The studies were performed with 10-week-old male Wistar rats from the Mossakowski Medical Research Institute, Polish Academy of Sciences, Warsaw, Poland. The animals were kept in groups of two or three per cage and maintained on a 12 h/12 h light/dark cycle with free access to a standard pellet diet and tap water. The floor area provided per animal was at least 350 cm^2^, accounting for potential weight gain. The animals were randomly divided into two groups: control and diabetic groups. Each experimental group consisted of 5 rats, as the albumin permeability values for isolated glomeruli from control and diabetic animals are 0.192 ± 0.171 and 0.595 ± 0.135, respectively (the mean ± SD). Considering the significance level α = 0.05 and test power 0.90 (http://biomath.info/power/ttest.htm, accessed on 30 October 2018), the calculated sample size is less than 6 rats for each group. Diabetes was induced by an injection of STZ (80 mg/kg, i.p.). Age-matched Wistar control rats were injected with a vehicle (citrate buffer). Fasting blood glucose concentrations were measured in whole blood samples using a glucose oxidase analyzer (Accu-Chek Performa, Roche Diagnostics, Mannheim, Germany). Glycemia > 250 mg/dL on day 3 post-injection was considered indicative of successful diabetes induction. No animals were excluded during these experiments. The rats were kept in separate metabolic cages (one rat per cage, Tecniplast, with a floor area of 320 cm^2^) for 48 h (day 15 to 17 after diabetes induction). The animals were first allowed to habituate to the cages for 24 h. Urinary albumin concentrations were determined in an external laboratory (Medical Laboratories BRUSS, Gdynia, Poland). The amount of urinary excretion per day and drinking water volume per day were calculated. On day 17 after diabetes induction, the rats were sacrificed to isolate their kidneys. Metabolic balance studies in healthy male Wistar rats (control) and Wistar rats with STZ-induced diabetes are presented in Table 1.

### 4.3. Reagents

The cell culture media and fetal bovine serum (FBS) were sourced from Thermo Fisher Scientific, Waltham, MA, USA. Antibiotics and trypsin were obtained from Merck, Darmstadt, Germany. Recombinant human Klotho protein (active) was purchased from Abcam, Cambridge, UK (catalog no. AB84072). The ADAM17 inhibitor *N*-(*R*)-(2-[hydroxyaminocarbonyl]methyl)-4-methylpentanoyl-L-naphthylalanyl-L-alanine amide (TAPI-0) was purchased from Merck (catalog no. SML1292).

### 4.4. Human Podocyte Cell Culture

Human immortalized podocytes were kindly provided by Prof. Moin A. Saleem from the University of Bristol. Undifferentiated cells were cultured at 33 °C in RPMI 1640 medium supplemented with 10% FBS, 100 U/mL penicillin, and 100 μg/mL streptomycin to promote proliferation until the desired confluence was achieved. To induce differentiation, the cells were placed at 37 °C, and the experiments were conducted on podocytes 10–16 days afterward. During the last 5 days of culture, the cells were grown with standard (SG; 11 mM) or high HG (30 mM) glucose. Additionally, the cells were treated with Klotho protein (0.5 nM) for the last 24 h or with TAPI-0 (10 µM) for 20 min.

### 4.5. Antibodies

The antibodies used for Western blot analysis were as follows: mouse anti-ADAM17 (catalog no. sc-390859, Santa Cruz Biotechnology, Dallas, TX, USA), rabbit anti-Klotho (catalog no. SAB3500604, Merck), and mouse anti-actin (catalog no. A3853, Merck). Anti-ADAM17 antibody was also used for immunohistochemistry. Horseradish peroxidase-conjugated secondary antibodies were obtained from Merck.

### 4.6. Western Blot

Protein from podocytes or glomeruli was extracted with a lysis buffer (1% Nonidet P-40, 20 mM Tris, 140 mM NaCl, 2 mM ethylenediaminetetraacetic acid, and 10% glycerol) that contained protease and phosphatase inhibitors as described previously [22]. Equal amounts of protein were separated by sodium dodecyl sulfate-polyacrylamide gel electrophoresis and transferred to polyvinyl difluoride membranes. The primary antibodies that were used in this study were diluted as follows: 1:800 for anti-ADAM17, 1:445 for anti-Klotho, and 1:10,000 for anti-actin. Horseradish peroxidase-conjugated secondary antibodies (1:3300) were used to detect the proteins. Densitometric band quantification was performed with Quantity One 4.6.3 software (Bio-Rad, Hercules, CA, USA).

### 4.7. Immunofluorescence

The specimens were imaged using a fluorescence microscope (Nikon Eclipse Ti-E, Nikon Instruments, Melville, NY, USA) with the Re-scan Confocal Microscopy module from Confocal.nl (Amsterdam, The Netherlands) [46] and a 60× oil immersion lens. Cells were immunostained following a previously established protocol [47]. Single staining was achieved by incubating the cells with primary anti-ADAM17 antibody (1:50, Santa Cruz Biotechnology) and Alexa Fluor 546-conjugated secondary anti-mouse antibody (1:400, Thermo Fisher Scientific). The negative control cells were stained without primary antibodies. The exposure time and all other settings (i.e., gain, gamma, and the intensity of excitation) were kept consistent across all samples, including the negative controls. The corrected total cell fluorescence (CTCF) was measured using ImageJ 4.1 software (National Institutes of Health, Bethesda, MD, USA) with the following formula: CTCF = Integrated Density—(Area of selected cell × Mean fluorescence of background readings). The F-actin network was visualized by fluorescence microscopy (Nikon Eclipse Ti-E) following the method described previously [48]. Actin was stained using Alexa Fluor 594 phalloidin (1:200, Thermo Fisher Scientific). Digitized fluorescence images of the F-actin network were used to generate fluorescence intensity profiles (from the basal membrane to nucleus) using NIS-Elements 5.11.02 imaging software.

### 4.8. Immunohistochemistry

Kidneys were dissected from control and streptozotocin (STZ) Wistar rats. The immunohistochemistry protocol was performed as described previously [30]. In brief, kidney sections were deparaffinized and rehydrated by washing with Histochoice Cleaning Agent (Merck) followed by a series of ethanol solutions (100%, 95%, 90%, 70%, and 50%). Antigen retrieval was performed by heating the slides in citrate buffer (10 mM, pH 6). The slides were then blocked with 5% bovine serum albumin (BSA) in phosphate-buffered saline (PBS) and incubated overnight with the primary anti-ADAM17 antibody (1:20, Santa Cruz Biotechnology). The next day, the slides were incubated with secondary antibody (Cell Signaling Technology, Danvers, MA, USA). Subsequently, the slides were incubated with Signal Stain DAB Substrate (Cell Signaling Technology) for 2 min followed by immediate washing in double-distilled water. The samples were counterstained with hematoxylin and dehydrated in ethanol and Histochoice Cleaning Agent. Tissue sections were imaged with a light microscope (Eclipse Ti; Nikon Instruments, Melville, NY, USA). Quantification of the amount of stained proteins was performed using ImageJ 4.1 software.

### 4.9. ADAM17 Activity Assessment

The proteolytic activity of ADAM17 in biological samples was measured using the optimized substrate ABZ-Asn-Tyr-Met-Ala-Leu-Arg-Arg-Lys(Dnp)-NH_2_ [26]. The reactions were performed as follows. Initially, 10 µL of cell lysate or extracellular medium and 170 µL of assay buffer (25 mM Tris-Cl, 2.5 µM ZnCl_2_, and 0.005% Brij-35 [pH 9.0]) were added to each measuring well and mixed with 20 µL of the substrate (at a final concentration of 3.74 × 10^−5^ M in the test well). As before, 10 µL of cell lysate or cell supernatant was added to each measuring well, supplemented with 160 µL of assay buffer. After adding 10 µL of the inhibitor (final concentration in the test well: 1.00 × 10^−6^ M), the system was incubated for 30 min at 37 °C. Afterward, 20 µL of the substrate ABZ-Asn-Tyr-Met-Ala-Leu-Arg-Arg-Lys(Dnp)-NH_2_ was added, and fluorescence was measured using a CLARIOStar microplate reader (BMG LABTECH, Ortenberg, Germany) for 30 min. In the case of rat urine, 40 µL of biological sample, 10 µL of inhibitor, 130 µL of buffer, and 20 µL of substrate were used. Urine probes were normalized by the volume of daily collection.

### 4.10. Lentivirus-Derived Stable Gene Silencing

Lentiviral shRNA particles (GIPZ shRNA Viral Particle Starter Kit, Horizon Discovery, Waterbeach, UK) were utilized to establish a human podocyte cell line with stable silencing of the *ADAM17* gene (catalog no. VGH5526-EG6868, clone ID: V3LHS_322733, shADAM17 podocytes) and *Klotho* gene (catalog no. VGH5526-EG9365, clone ID: V3LHS_361651, shKlotho podocytes). The cells that were infected with “GIPZ Non-silencing Lentiviral shRNA Control” particles (a part of the sets) served as the control group (shCtrl podocytes). Gene silencing was carried out according to the manufacturer’s instructions. To eliminate uninfected cells (which lacked puromycin resistance), three rounds of puromycin selection (0.8 µg/mL) were performed.

### 4.11. Quantitative Real-Time Polymerase Chain Reaction

Total RNA was extracted from cultured podocytes as described previously [30] using the RNeasy Mini Kit (Qiagen, Hilden, Germany), which included an on-column DNase treatment step using the RNase-Free DNase Set (Qiagen). The concentration and purity of RNA were determined using a NanoDrop spectrophotometer (Thermo Fisher Scientific). Subsequently, the isolated RNA underwent reverse transcription, and the levels of specific mRNA transcripts were quantified through real-time polymerase chain reaction (PCR) using a LightCycler 480 (Roche Diagnostics). Gene-specific intron-spanning primers and fluorescent hydrolysis probes were employed in the PCR analysis. The resulting amplified products were separated in 2.5% agarose gel and visualized using the GelDoc-It Imaging System (Ultra-Violet Products, Mile End South, South Australia). The sequences of the primers and probes are provided in Table 2.

### 4.12. Measurement of Intracellular ROS Levels

Reactive oxygen species production was measured with the fluoroprobe 2′,7′-dichlorodihydrofluorescein diacetate (H_2_DCFDA) as described previously [18]. Briefly, podocytes were incubated in PBS that was supplemented with appropriate concentrations of glucose (SG or HG) for 2 h; then, 10 μM H_2_DCFDA was added for the next 30 min; and cells were incubated at 37 °C in an atmosphere of 95% air and 5% CO_2_. H_2_DCFDA was quickly absorbed by the cell and converted by intracellular esterases into 2′,7′-dichlorodichydrofluorescein (DCF). To prevent visible light-induced degradation of DCF, the cells were incubated in the dark. The cells were washed twice with PBS, and the fluorescence emission of DCF was immediately measured using a spectrofluorometer (LS55, Perkin Elmer, Waltham, MA, USA) at excitation and emission wavelengths of 485 and 525 nm, respectively.

### 4.13. NADPH Oxidase Activity Assay

NADPH oxidase activity was measured by lucigenin-enhanced chemiluminescence as described previously [49]. The amounts of superoxide were calculated by integrating the area under the signal curve. These values were compared with a standard curve that was generated using xanthine/xanthine oxidase, as published previously [50].

### 4.14. Isolation of Glomeruli

The protocol for the isolation of glomeruli was performed as described previously [30]. Briefly, the kidneys were excised and placed in ice-cold PBS (pH 7.4) that was supplemented with 0.49 mM MgCl_2_, 0.9 mM CaCl_2_, and 5.6 mM glucose. The renal capsule was removed, and the cortex was minced and pressed through a series of sieves with decreasing pore diameters (250, 125, and 75 μm). The resulting suspension contained decapsulated glomeruli. The entire procedure was carried out on ice and completed in under 1 h.

### 4.15. Glomerular Permeability Assay

The volume response of glomerular capillaries to an oncotic gradient that is generated by changing concentrations of albumin was analyzed as described previously [51,52]. The volume response of glomerular capillaries to an oncotic gradient, created using defined concentrations of albumin, was measured. Briefly, isolated glomeruli were fixed on poly-L-lysine-coated glass coverslips and incubated in a medium that contained 5% BSA and the ADAM17 inhibitor TAPI-0 (10 µM, 20 min) at 37 °C. The glomeruli were then washed with 5% BSA medium to remove the ADAM17 inhibitor. Finally, the glomeruli were placed in a medium that contained 1% BSA to generate an oncotic gradient across the capillary wall, while control glomeruli were incubated in fresh 5% BSA medium (without the oncotic gradient). Changes in glomerular volume (V) were recorded by videomicroscopy (Olympus IX51) and calculated according to the following formula: V = [4/3A$\sqrt{A/\pi }$]/10^6^, where A is the glomerular surface area (calculated with CellSens Dimension 1.18 software, Olympus, Hamburg, Germany). Volume changes (ΔV) were calculated using the formula ΔV = (V_final_ − V_initial_)/V_initial_, where the increase in ΔV is directly related to the oncotic gradient applied across the capillary wall. This principle was used to calculate the reflection coefficient of albumin (σ_alb_), defined as the ratio of ΔV for experimental glomeruli to ΔV of control glomeruli in response to identical oncotic gradients, where σ_alb_ = ΔV_experimental_/ΔV_control_. The reflection coefficient of albumin (convectional P_alb_) was used to calculate glomerular capillary permeability to albumin (1 − σ_alb_), which describes the movement of albumin consequent to water flow.

### 4.16. Transepithelial Permeability Assay

Transepithelial permeability to albumin was evaluated by measuring the diffusion of FITC-labeled BSA across the podocyte monolayer as described previously [30]. In short, for this experiment, podocytes were first cultured on cell culture inserts that were coated with type IV collagen. Before the experiments, the medium was replaced with serum-free RPMI 1640 (SFM) for 2 h. Afterward, the medium in the upper chamber was replaced with fresh SFM, while SFM was added in the lower chamber with fluorescently labeled (FITC) BSA at a concentration of 1 mg/mL. After 1 h, the solution from the upper chamber was collected and transferred to a 96-well plate, and the absorbance of FITC-BSA was measured at 490 nm using an ELx808 Absorbance Reader (BioTek Instruments, Winooski, VT, USA).

### 4.17. Statistical Analysis

The data were analyzed using GraphPad Prism 5.03 software. Normality of the data distribution was assessed by the Shapiro–Wilk test. Based on the results of this test, statistical significance was determined using either a parametric test (one-way analysis of variance [ANOVA]) or a nonparametric test (Kruskal–Wallis test), followed by an unpaired t-test or the Mann–Whitney test, as appropriate. The results are expressed as the mean ± SD. Values of *p* < 0.05 were considered statistically significant.

## Figures and Tables

**Table 1:** Metabolic balance studies in control and diabetic Wistar rats.

Parameter	Control Rats (*n* = 5)	STZ Rats (*n* = 5)
Body weight (g)	367.6 ± 44.3	255.6 ± 42.1 **
Urine volume (mL/24 h)	14.18 ± 1.72	217.8 ± 30.1 ****
Blood glucose concentration (mg/dL)	168.8 ± 32.83	497.0 ± 66.7 ****
Urinary albumin excretion (mg/24 h)	0.17 ± 0.07	4.15 ± 2.75 *

The data are expressed as the mean ± SD. * *p* < 0.05, ** *p* < 0.01, **** *p* < 0.0001, vs. control rats.

**Table 2:** Primers and probes used for real-time PCR.

Gene	Accession No.	Primer Sequences	Probe Sequences	PCR Product Length [bp]
*ADAM17*	NM_001382777.1	Forward: TTTGTGGGAACTCGAGGGTG Reverse: GCACTTCTTCTGGGCAGTCT	ACCTGCTG	173
*Klotho*	NM_004795.4	Forward: GCTCAACTCCCCCAGTCAGG Reverse: TGTGGGCTTTGAGAGCTTCG	CCAGGGCA	193
*ACTB*	NM_001101.5	Forward: ATTGGCAATGAGCGGTTC Reverse: GGATGCCACAGGACTCCA	CTTCCAGC	76

*ACTB*, gene coding β-actin; PCR, polymerase chain reaction.

## Data Availability

The original contributions presented in this study are included in the article. Further inquiries can be directed to the corresponding author.
